# Work as a social determinant of maternal health: A qualitative exploration of college-educated Black women’s experiences at work during pregnancy and postpartum

**DOI:** 10.1177/17455057241304842

**Published:** 2024-12-04

**Authors:** Serwaa S Omowale, Laurenia C Mangum, Andrea Joseph-McCatty, Cherell Cottrell-Daniels, Kaiya A Farris, Rashon King, Brittany C Slatton

**Affiliations:** 1 Department of Management, Policy, and Community Health, School of Public Health, University of Texas Health Science Center at Houston, Houston, TX, USA; 2 School of Social Work, University of Pittsburgh, Pittsburgh, PA, USA; 3 Jane Adams College of Social Work, University of Illinois Chicago, Chicago, IL, USA; 4 College of Social Work, University of Tennessee, Knoxville, TN, USA; 5 Health Choice Network, Miami, FL, USA; 6 Department of Health Promotion and Behavioral Sciences, School of Public Health, University of Texas Health Science Center at Houston, Houston, TX, USA; 7 School of Public Health, University of Texas Health Science Center at Houston, Houston, TX, USA; 8 Department of Sociology, Texas Southern University, Houston, TX, USA

**Keywords:** determinants, health status disparities, maternal health, pregnancy, postpartum, psychological stress, discrimination, work

## Abstract

**Background:**

Black women are disproportionately impacted by higher rates of maternal mortality in the United States (US). Limited research has focused on adverse maternal health outcomes among college-educated Black women, although research has found these outcomes persistent among this population.

**Objectives:**

This study aimed to fill a critical gap in current research by elucidating the nuanced experiences of college-educated Black women in the workplace during pregnancy and postpartum. By exploring this under-researched area, our study contributes to the academic discourse on Black maternal health disparities within the scope of occupational health. It offers practical insights for enhancing workplace gender equity, informing healthcare practices, and shaping policies that support equitable maternal health outcomes among Black women.

**Design:**

This qualitative study conducted semi-structured interviews with 17 Black mothers between March 2019 and September 2019.

**Methods:**

Seventeen in-depth interviews were conducted with college-educated Black women in the US from March 2019 to September 2019. Participants were asked several questions about work stress, discrimination, and other workplace experiences during pregnancy and postpartum.

**Results:**

Our analysis identified three themes, including Strong Black Woman/Superwoman, work stress (sub-themes: consciousness of work stress, mental and physical responses to stress, and work–family conflict), and perceived work-related discrimination.

**Conclusion:**

For college-educated Black women experiencing pregnancy and postpartum, it is crucial to understand the structural and social determinants of optimal occupational health. It is critical that workplaces enact occupational health equity with attention to racial, gender, and disability-centered considerations to address the unique challenges experienced by Black women.

## Introduction

Black women are disproportionately impacted by higher rates of maternal mortality in the United States (US).^1^ In 2022, maternal mortality (deaths that occur while pregnant or within 42 days of termination of pregnancy) was the highest among Black women (49.5 deaths per 100,000 live births).^2^ Among college-educated Black women with bachelor’s degrees or higher, pregnancy-related deaths (2007–2016) were five times greater than their white women counterparts.^3^ Higher maternal education has been considered a low-risk group for adverse pregnancy outcomes, but college-educated Black women have not gained the same maternal health advantage as other women. Therefore, assumptions about education, class, and resources as health protective factors are not always applicable to college-educated and middle-class Black women without understanding the intersectionality they experience related to race, gender, and class oppression.^4,5^ Hence, factors contributing to adverse pregnancy outcomes among college-educated Black women need to be examined to understand better the determinants impacting maternal health in this population.

Work as a social determinant of health and its impact on maternal health outcomes has been under-researched.^6,7^ The role of work in maternal and child health literature has been limited to the impact of work on breastfeeding^8–11^ and paid maternal/family leave disparities.^12–15^ In the US, work in the form of occupation and salary determines your housing/neighborhood environment, access/quality of healthcare since healthcare is linked to employment, and food options, which are all factors that influence health. One factor contributing to adverse health among employees is work-related stress, also called job strain or job stress. Work-related stress encompasses job demands (e.g. workload, other stressors) and decision latitude (discretion or control) in deciding how to meet these demands. Job strain is caused by high job demand and low decision latitude.^16^ Also, work-related stress is triggered by a person’s appraisal of their situation at work, their inability to cope with work demands, insufficient control over their situation at work, and a lack of support to cope with these stressors in the work environment.^17^ Furthermore, inadequate response to threatening stressors can lead to adverse physiological and psychological outcomes.^18,19^ Therefore, stress related to work may have implications for maternal health.

## Theoretical framework

Black feminist theory (BFT) is a foundational framework for understanding how Black women navigate and resist intersecting oppressions of race, class, and gender.^20^ Originating in the 1970s and 1980s, BFT critiques the lack of attention to Black women’s experiences in both feminist and civil rights movements, advocating for an inclusive analysis that addresses the multiplicity of oppression.^20,21^ BFT asserts that Black women’s lived experiences are marked by a unique intersection of race, gendered, and class-based oppression, which cannot be fully understood through the analytical lenses of traditional feminist or race theories alone.^20,21^

One of the core concerns of BFT is the exploitation of Black women in the places they labor and work. The historical context of Black women’s work and labor in the US is rooted in oppression that includes exploitation of their labor, sexual violence, medical experimentation, the commodification of their bodies for profit during the enslavement of African women in the US, and later exploitation as domestic workers for White families.^22–24^ Contemporary career and employment opportunities have expanded in the job market for Black women, especially for the college-educated and highly skilled. However, discrimination against Black women persists in the workplace, which involves unfair work demands, stereotypes, isolation at work, perceived incompetence, harassment, and wage disparities.^25–28^

By integrating BFT, our theoretical framework places the complex, intersectional experiences of Black women at the forefront of analysis, ensuring a comprehensive understanding of their unique challenges. This framework offers a critical lens to understand the unique challenges faced by pregnant and postpartum Black women in the workplace, explaining how discrimination, stereotyping, and unfair labor practices amplify stress and further deepen the health disparities within this group, such as higher rates of maternal mortality and morbidity.

## Purpose of study

Guided by a phenomenological inquiry, The *Black Women’s Work and Maternal Health Study* aimed to fill a critical gap in current research by elucidating the nuanced lived experiences of college-educated Black women in the workplace during pregnancy and postpartum. We utilized BFT to design the study interview guide, recruitment, data collection, and data analysis. By exploring this under-researched area, our study contributes to the academic discourse on Black maternal health disparities and occupational health. It offers practical insights for enhancing workplace gender equity, informing healthcare practices, and shaping policies that support equitable maternal health outcomes.

## Methods

### Participants

This study included seventeen self-identified Black or African American women who met the following inclusion criteria: (1) possession of at least a 4-year college degree or higher, (2) born in the US, (3) had a live birth within the past 24 months, and (4) employment (full or part-time) during or before their most recent pregnancy. We excluded pregnant women from the study because one of the objectives was to understand the postpartum experiences of participants. Additionally, Black women born outside the US were excluded based on previous research indicating that foreign-born Black immigrants generally have better birth outcomes compared to their native-born counterparts.^29^

### Recruitment

We recruited participants primarily through social media, targeting Facebook groups likely to reach our study population, such as graduate chapters of African American sororities, Historically Black Colleges and Universities, moms, and Black breastfeeding groups. Additionally, we collaborated with maternal and child health organizations like The Allegheny County Infant Mortality Collaborative and the IMPACT Collaborative in Houston, Texas, distributing our study flyer across their networks. Referrals from Black women with young children to others within their social circles emerged as the most effective recruitment strategy, and convenience snowball sampling was used. Participants verbally consented to participate in the study before completing the demographic survey and study interview. Verbal consent was taken to make participant responses anonymous due to the sensitive nature of participants discussing employment/employers and birthing experiences (i.e. birth trauma). The final sample size of 17 was determined after data saturation was reached at 17 participants.^30,31^ Each participant was assigned a participant ID, and names were not linked to their background survey and study interview. Each participant received a $25 retail store gift card as compensation for their contributions and a handwritten thank you card after the in-person interview. The University of Pittsburgh Human Research Protection Office approved this study (IRB Number # STUDY1902029).

### Data collection

Prospective participants were screened for eligibility. Next, participants were sent a demographic survey via email using a University of Pittsburgh approved Qualtrics survey. The principal investigator (PI, author S.S.O.), a college-educated Black woman, conducted all semi-structured interviews, offering participants various options for interview settings, including their workplaces, local library private rooms, apartment clubhouses, and homes, to minimize participation barriers. Encouraging participants to bring their young children to the interviews further reduced childcare barriers, allowing many to tend to their infants or toddlers during the process. These measures aimed to lower research participation barriers and prioritized the parenting experiences of Black women. Data were collected from March 2019 to September 2019; on average, participants completed the study within 1–2 months, depending on their availability to complete in-person interviews. The Consolidated Criteria for Reporting Qualitative research (COREQ) was used when preparing this manuscript.^32^

### Measurement

In this study, phenomenology was used alongside BFT to prioritize and understand the lived experiences of individuals, particularly those who are marginalized.^20,33,34^ A phenomenological approach was most suitable for this study as it enabled the in-depth exploration of Black women’s perspectives and meaning making on work and mental health during pregnancy. Similarly, BFT provided the critical lens to examine how intersecting oppressions shaped their lived experiences. Guided by BFT we developed a 20-question interview guide, reflective of Black women’s lived experiences, and through a structured three-step process. We conducted an exhaustive review of BFT literature, covering both its historical roots and contemporary developments. This foundational step informed our understanding of the framework’s relevance to our study’s focus areas. Next, we integrated specialized knowledge based on the African American women’s standpoint, a concept articulated by Patricia Hill Collins.^34^ BFT recognizes that Black women have their own epistemology, which is reflected in the “Black woman standpoint.” Collins^34^ states that Black feminist scholars should bring the experience of everyday Black women into the academy. This approach allowed us to incorporate insights derived from the lived experiences of Black women outside the academic sphere directly into our interview questions. Finally, we refined our instrument by soliciting feedback on the draft interview guides from our co-author (L.C.M.) and the Maternal Health Equity Scholars at the University of Pittsburgh, ensuring that our questions were relevant and sensitive to the nuances of our participants’ experiences. The finalized questions addressed various aspects of the participants’ lives, including workplace stress, discrimination, policies on work leave, and adjustments upon returning to work postpartum. We also explored broader topics such as maternal health, birth outcomes, and postpartum experiences. Interview guide questions included (1) *How would you describe your role in your family? How does this influence your work experience?* and (2) *Have you experienced any incidents at work you believe are due to discrimination?* See Supplemental Appendix 1 for full interview guide.

### Data analysis

All interviews were audio recorded and transcribed verbatim by a professional transcription service, and later reviewed and edited for accuracy by the PI. All the final transcripts were transferred into Dedoose (v9.2.4.; Los Angeles, CA, SocioCultural Research Consultants, LLC) software to organize coding. This study used Braun and Clarke’s^35^ methods of reflective thematic analysis. The PI and research team (authors K.A.F. and R.K) completed the data analysis. Before the analysis, the PI developed priori codes based on work-stress and discrimination theory (e.g. work-related stress, work-related discrimination). This codebook was used for the initial coding by the team; over the first 3 cycles of coding, codes, and sub-codes were added and deleted to formalize the final codebook. Team members conducted coding independently, coding one transcript at a time, and used an open coding approach.^36^ During weekly team meetings, the research team would discuss coding challenges, discrepancies, emerging codes, and analytical memos.^37^ Additionally, we used data visualization tools, code co-occurrence, and code application per file to establish confirmability. The final coding (cycle 4) was used to conduct the thematic analysis, and the research team and co-authors agreed on the final themes and exemplary quotes.

### Positionality

The research team that conducted this data analysis comprised three Black women (authors K.A.F., R.K., and S.S.O.). The analysis team met weekly to facilitate opportunities for reflection and ultimately foster dependability and confirmability through the analytic process. During data analysis meetings, the team reflected on their lived experiences as Black women who work outside the home and how these experiences could influence our interpretations of the data. It was important that we were reflective as a team because each team member expressed similar experiences indicated by some of the participants as mothers who work during pregnancy and postpartum (K.A.F. and R.K). Our reflective practice during the analysis process drew on BFT in its understanding and interpretation. Black feminist scholar Patricia Hill Collins^20^ states, “Black feminist thought encompasses theoretical interpretations of Black women’s reality by those that live it.” Moreover, our team used our lived experiences to understand the data better, and we also challenged our assumptions through this group’s reflective process.

## Results

### Sociodemographic

The study sample comprised 17 Black women who self-reported a live birth from 2017 to 2019, with the majority giving birth in 2018 (*n* = 8). Participants identified as African American (*n* = 7), Black American (*n* = 3), Black (*n* = 6), African American and Nigerian American (*n* = 1). Most participants were married (*n* = 16) and first-time moms (*n* = 10). The average age was 35 years old; 47% of the participants reported a yearly income of $60,000 or more, and the average worked 40 h a week. On average, participants started prenatal care at 7 weeks of gestation. Table 1 details the participant demographic information, including educational level, occupation, and state residency.

**Table 1.** Participant demographic information.

Pseudonym	Age	Education	Occupation	Hours per week at work	Participant residency	Marital status	Income range
Tina	37	Masters	Professor/Researcher	46	Texas	Married	more than $60,000
Gina	37	Masters	Case management	50	Texas	Married	more than $60,000
Maya	36	Masters	Registered nurse	40	Maryland	Married	more than $60,000
Mia	42	Masters	School counselor	35	New Jersey	Single	more than $60,000
Mary	35	Masters	Quality improvement project manager	46	Virginia	Married	more than $60,000
Briana	29	Masters	Higher education professional	40	North Carolina	Married	$45,000–$60,000
Toya	29	Masters	Social worker/care coordinator	43	Virginia	Married	$45,000–$60,000
Chole	37	Masters	Educator–Administrator	15	North Carolina	Married	$15,000–$30,000
Jada	38	Bachelors	Stay-at-home mom	0	North Carolina	Married	0–$15,000
Kayla	28	Bachelors	Admin Assistant	37	North Carolina	Married	$30,000–$45,000
Imani	29	Masters	Social work	40	North Carolina	Married	$45,000–$60,000
Nia	33	Bachelors	Healthcare executive director	50	North Carolina	Married	more than $60,000
Tia	36	Masters	Teacher/director	50	Georgia	Married	$30,000–$45,000
Aba	35	Masters	Program manager	40	Louisiana	Married	more than $60,000
Afua	38	Masters	Communications specialist	44	Georgia	Married	$45,000–$60,000
Susie	39	Doctorate	Lawyer/entrepreneur	47	Texas	Married	more than $60,000
Racheal	29	Bachelors	Lifeguard	24	Pennsylvania	Married	$15,000–$30,000

## Findings

Our analysis identified three themes: Strong Black Woman/Superwoman, work stress (sub-themes: consciousness of work stress, mental and physical responses to stress, and work–family conflict), and perceived work-related discrimination.

### Theme 1 Strong Black Woman/Superwoman: “There’s no place in this world for a crying, Black lady”

Extant literature on Black women’s coping with stressors has documented the phenomenon of Strong Black woman or superwoman schema (SWS).^38–40^ This schema is often considered a protective factor, a coping resource, and enables Black women to have positive coping responses to the stressors and stimuli that are experienced instead of developing avoidant behaviors. What is counterintuitive with this phenomenon is that woman can endure greater stress, adversities, and problems because of an inanimate and superficial belief that their bodies can endure the stresses. Many of the participants described their role in their family when asked as, “I am the breadwinner,” “the CEO,” and “wife, mother.” Additionally, they detailed the support they provided to their nuclear family and, in many cases, extended family members. Moreover, several expressed the need to personify strength within their family, careers/educational goals, and work/employment. Additionally, they reflected on the expectations of them as Black women.

One participant stated her understanding of the African American cultural expectation of Black women as the following:

So I feel like in the African American culture—you know—the woman is looked at as, “We have to take care of everything.” And I also feel like society places a lot of responsibility on the woman. Yeah. The man has the things he’s supposed to do for his family, but I feel like society in the African American culture, men are looked as the provider. And when I say provider, monetarily. The woman is supposed to do everything else.

Another participant reflected on the cultural expectation of being a superwoman in her relationship with her husband as the following:

“I had to get on him because he’s always saying, You’re superwoman.” I’m like, “I’m not. Don’t call me that. Don’t call me superwoman because then you—, because then just that I feel like negates all the human things that I experience and that I go through and then that it can just kind of help me neglect those things, and that’s not okay. So I’m always on because he continues to do that. “Oh, you’re superwoman. You can do everything, no. And so that’s always like a constant back and forth with us.”

Many of the participants conveyed the need to be strong as they worked to achieve their career goals and education. Furthermore, they expressed the intersectionality of race, gender, and society on this cultural norm. One participant expressed how exemplifying strength is a cultural norm for Black women:

There’s no place in this world for a crying, black lady. A black girl with tears. There’s no room for us here. At no point in life do you ever see black girls cry and move ahead. That’s not what happens. That’s a luxury that our melanin does not provide us. . . Because there’s no room for our tears . . . You got to be tough. . . . You got to be strong. So that’s who I am. That’s what I am, and that what most of us are. So yeah. That’s it. It’s in our DNA.

Another participant reflected on how the intersection of race, gender, and age in society influences her strength in the workplace:

As far as being a young black female, it can be challenging. I feel like we have to work 10 times harder to prove ourself and then being a female alone, male versus female. Before this role I was a regional director for a food management company, and it’s a predominantly male-driven industry. And breaking down those barriers to get where I needed to be, that took a lot of adversity, a lot of strength. And like I said, I had to work 10 times harder because I was a female, and then I was a black female. So, breaking down those barriers was challenging, but I did it.

Within this context, the strength that participants described can best be explained by the “Super Woman Role” (SWR) concept. Black feminist scholars such as Beauboeuf-Lafontant^38,39,41^ have explored this notion of strength within their work. The SWR has emerged as a coping strategy utilized by Black women, emphasizing strength as a form of resistance that has both positive and negative psychological implications on health.^42^ In sum, the Black women in this study both embraced being strong women as a form of resistance against gendered racism in society and to achieve professional goals. However, some participants rejected this cultural expectation within their families to exhibit strength.

### Theme 2 Work-related stress: “I have a baby in my belly. I will not let this job cause me a miscarriage”

Work-related stress can be attributed to role strain within the workplace in addition to factors associated with the workplace such as the actual environment, leadership and management, relationships with colleagues, and the act of preparing oneself to travel to work (traffic, transportation needs, etc.). The occupational environment contributes to the overall wellness of its employees. When we consider the multiple minoritized identities of Black women, in addition to their roles as primary caregivers in the home, Black women experience multiplicative stress different from other racial/ethnic working women who are also caregivers. Narratives from the participants revealed experiences of work-related stress over the course of their career/employment as adults, during pregnancy, and postpartum. We identified three of the following sub-themes related to work stress during pregnancy and postpartum: (1) consciousness of work stress, (2) mental and physical responses to stress, and (3) work–family conflict.

#### Consciousness of work stress

Several participants conveyed being conscious of work stress during pregnancy and attempts to navigate work and pregnancy. Moreover, participants attempted to mitigate work stress, but often it was unavoidable due to the nature of their positions. One participant was aware of the potential stress she would experience if she accepted a disaster management role during a weather disaster in her city. She stated, *“Well, then they asked me to manage a DRS team, disaster recovery team for seniors, and I was like, “No.” Because I came from disaster recovery, so I knew what the stress was going to be like.”* The participant explained that she was repeatedly pressured to take on this role even though her direct supervisor knew she was pregnant. The participant eventually accepted the role. Another expressed how she was aware that work stress was impacting her medical vitals during pregnancy and her provider suggested that she pull back from work during pregnancy. She expressed the following about work stress and her pregnancy:

When they realized the amount of stress. So, the funny—it’s not a funny story, but the funny story is that when I would go to appointments in the morning, everything would be pristine. If I came in at 2 o’clock, it looked like I was a high-risk pregnancy. And so maybe 6 or 7 months into the pregnancy, they did ask me to pull back. I did start working from home more, but I was still going in the way of working. So, I am more calm when I’m not around the 45 people that I work with every day, but I did keep the intensity. [Worked in corporate setting].

#### Mental and physical response to work stress

Many of the participants reflected on work stress during pregnancy and postpartum and how that impacted their mental and physical health during these periods. One participant that was a teacher stated, *“And so—I was sickly and in the classroom, so there wasn*’*t flexibility around when I could come into work and when I couldn*’*t because I had to be there for children and be in place for them. It was very physically demanding. I was sick all the time throughout the entire pregnancy. I probably didn*’*t sleep enough. I wasn*’*t taking prenatals enough. I wasn*’*t drinking enough water. It was very stressful.”*

Many participants described the stress of returning to work postpartum and the impact that had on their mental and physical health. Two participants described the following experiences returning to work:

“So I feel like I was most stressed when I came back to work after being on maternity leave.” Because I came back in the middle of admission season, and I’m on the admissions committee. So, I was tasked with reading 80 applications,—plus interviewing all of the candidates who had applied to our part-time program. And so, during that time, when I would come home, I would just feel so, so stressed. And then figuring out parking and transportation, I was feeling very stressed and overwhelmed. So, I would come home and just play with the baby. I really had no other coping mechanism or self-care routine. [Worked in higher education].

My job, for whatever reason, just decided, “Oh, I can’t—” teleworking anymore—and for me, my telework is a part of a reasonable accommodation, so it’s medically necessary. And just the fight to have to get that back, it really started affecting not only my physical health, but my mental health. I started having issues that I had never had, given everything I had already been through before. [Nurse].

Another participant described the physical response to work stress as the following:

Stress comes for me in the form of—internal stress I’ll say comes to me in the form of back spasms and tightness in my neck and chest and shoulders and sometimes like a nauseous type deal that’s not quite nausea. This unsettling of the spirit in your stomach. And so, I felt that numerous times in the last seven months, I think, that I’ve been back to work, and some before that because of the nature of the job and the nature of the expectation and the changes that take place.

#### Work–family conflict

Many of the participants expressed that returning to work postpartum was the most challenging. Several of them described prepping work tasks before maternity leave and for the expected changes in their family role with a new baby. However, many stated concerns around “work–life balance.” One participant that was a nursing home administrator with the responsibility of a geriatric population stated the following:

At times it can be stressful because my family doesn’t understand the level of responsibility here at work. They don’t understand that I can’t just clock out at 5:00 and be home. It doesn’t work like that. You’re going to have good days and you’re going to have bad days. And there are some things that I have to decide if I’m going to make or not.

Another perspective about work postpartum was changing positions or reducing the amount of additional work such as “side hustles,” “gig work,” or an outside business due to the pressures of managing work and family. The following participant decided to leave her job due to the challenges she found trying to manage both:

“Okay. I’m back at work, and all those demands that the family had are still there plus some because I have this super-tiny little human who is also counting on me for, I mean, literally everything,—nourishment [laughter].” And so, the responsibilities that I had from my family just grew, and also the responsibilities from work kind of just exploded. And so that is why I’m not working in that job anymore because I realized that I couldn’t, that both aspects needed so much of me that neither was getting a decent chunk, and that I couldn’t continue like that. And obviously, my family won.

Overall, participants expressed challenges with managing the new responsibilities of parenting and the demands of work. Also, many of the participants were in leadership roles or professional “White collar” jobs due to their college education, which may require more responsibility in their organizations and limited backup support during pregnancy and postpartum.

### Theme 3 Perceived discrimination: “We can’t have the Black girl who’s too ‘aggressive’ in that position”

Racial-gendered discrimination among Black women in the workplace presents as a function of poor workplace environments and a threat to optimal occupation health outcomes. Known adverse outcomes of perceived discrimination include stress, isolation and alienation, and unsatisfactory role performance. In addition, this can impact one’s self-identity and efficacy. Participants were asked about their experiences with discrimination in the workplace. None of the participants revealed any experiences of overt discrimination, but often they perceived discrimination. Moreover, they shared stories about past and current experiences at work. There were mixed responses regarding factors or why (e.g. race, gender) they perceived discrimination, often based on their work environment. For example, participants who worked with a majority of White women perceived race as a factor for discrimination. In cases where men were present in the work environment, gender and race were factors they felt contributed to discrimination. Some expressed that being a Black woman was the reason for discrimination. Often, these were experiences of being overlooked for promotions, undermining supervisory roles, microaggressions, and negative experiences with consumers. One of the participants expressed feeling “microaggressed against” after repeatedly being confused for another Black woman in her workplace. Several of the participants discussed entering their field after college or graduate school at a young age. A few participants expressed perceived discrimination due to race, gender, and age. The following participant shared this experience:

I work in healthcare, and the nature of my work in just my position as a project manager in quality improvement, all of the people who are in leadership positions are white, and the majority of them are men. So, when I’m having to do certain projects, trying to manage certain projects, I’m working with white men. Here’s the extra layer; they’re physicians, so there’s a bit of ego that I have to deal with. And so not only do I run into some of these microaggressions or discrimination, not just my gender because I’m a woman working with these men who are in positions where their ego has gotten pretty huge, but I’m also black. Second layer. But there’s a third layer, and that’s ageism. I think some of them treat me a certain way, speak to me a certain way because of my gender, my age, and my race.

Several participants felt they were overlooked for promotions. A few expressed, *“I feel like I had to work harder to prove myself”* or “*In the beginning when I was a bit more fresh in my career, I took it as you got to pay your dues and it comes with the territory. However, after we started getting into I have X-teen years worth of experience, it started to become frustrating for sure.”*

The following two participants shared their experiences of being overlooked for advancement at work:

“I’ve worked with majority Caucasian American individuals. And so regardless of my education, my experience, of my tenure, of my enthusiasm, of my passion, a lot of little things, little things that build up to big things, projects, opportunities, etc., would typically go to individuals who look like them. And then when asked—”Why am I not getting this?” but saying words like, “How can I gain more opportunities?” ”Oh, you’re doing everything perfectly. We love exactly who you are.” “Okay. If I’m doing that, then why am I not getting these opportunities?”

I was looking to move into a position and continue as a lead teacher and was told that I would not be good as a lead teacher even though I had been operating in a co-lead position. And they put a teacher in there who is not strong and has issues with different things. And she’s just not a good teacher. And the parents were telling me she wasn’t a good teacher, and so that, I felt, was more of a, oh, she’s a white female. We can’t have the black girl who’s too “aggressive” in that position

Many of the participants in the study expressed that they avoided addressing their experiences of discrimination at work. If they addressed any concerns, they carefully crafted their responses with colleagues or leadership to avoid being viewed as “ aggressive” or seen as the stereotype of an “angry Black woman.” Furthermore, many participants stated that their job was a vital resource for their families.

## Discussion

This phenomenological inquiry sought to explore the experiences of Black women at work during pregnancy and postpartum. Guided by a deductive approach, study findings offer a more nuanced understanding of the work (occupational) experiences of college-educated Black women during pregnancy and postpartum. Narratives from participants indicated the need to be strong and how Black women embody strength within their families, education, careers, and the workplace. These findings are similar to other studies of college-educated middle-class Black women.^25,26,43,44^ Also, the role of work-related stress and discrimination was explored to expand our understanding of stressors that potentially impact maternal health.

We found this intersection of strength, education, and careers among Black women in the study. Participants implied a need to be strong and resilient in the work environment over the life course and during pregnancy and postpartum. The “ Strong Black Woman” or “Super Woman Role” has been articulated by Black feminist theorists and Black women studies scholars as a form of resistance to the intersectional oppression that Black women experience in the US.^45–47^ Consistent with phenomenological inquiries that elucidate participant meaning-making, our findings demonstrate how Black women construct and navigate their identities within the intersecting contexts of work, pregnancy, and postpartum life. Their narratives reflect a deep engagement with the “Strong Black Woman” schema, revealing how this social expectation informs their lived experiences of both resilience and vulnerability. However, many scholars have also documented how this form of resistance has negative consequences for Black women, such as poor mental health.^48–50^ Research on depression by Walton and Boone^51^ found that middle-class Black women felt they could not be vulnerable, especially since past generations of women in their families had to struggle. Health research scholar Woods-Giscombé developed the Superwoman Schema to address the specific manifestations of stress experienced by Black women and its impact on health outcomes. The SWS describes a set of behaviors and beliefs predicated on strength, independence, and an unwavering commitment to caregiving, even at the expense of personal health and well-being.^40^ The SWS framework examines the descriptive factors associated with the superwoman role, moreover examining the “preconceptions, contextual factors, benefits, and liabilities, and beliefs regarding how it influences health”.^40^ While this schema has been posited as a coping strategy for contending with systemic oppression, research indicates that it may contribute to increased stress levels, leading to adverse health outcomes such as hypertension and mental health issues among Black women.^40,52,53^

We also found that participants were conscious of work-related stressors during pregnancy and postpartum. They shared experiences of work-related stress that impacted their mental and physical well-being and often led to work–family conflict. Work–family conflict is a form of inter-role conflict in which role pressures from work and family domains are mutually incompatible to a degree.^54^ Participants attempted to mitigate work-related stress postpartum by prepping work tasks before maternity leave. The historical and current exploitation of Black women’s labor is a core concern of BFT. For example, Black women make less income than their counterparts and experience major wage gaps with White women and men.^55,56^, and historically were limited to specific occupations such as domestic work. It is important to recognize these conditions in order to intervene and reduce work–family conflict and work-related stress. Recent research demonstrates that mothers adopt a mother-first identity^57,58^ and often triage their resources to meet the family’s needs while neglecting their own needs. College-educated Black women can leverage their education to earn higher salaries and close wage gaps. These women can bring important economic resources to their nuclear and often times extended family members. There has been a historical and current income and wealth gap between White and Black families.^59–61^ Thus, college-educated Black women may experience pressures to maintain employment, especially high-paying jobs that contribute substantially to the family. Furthermore, this can potentially exacerbate conflicts between work–family and internal stress. These stressors, if unaddressed, can impact Black women’s health, especially maternal health outcomes.

Many participants in the study expressed perceived discrimination through their careers in the work environment based on race, gender, and age. Many of the women in the study expressed experiencing stereotypes based on race, gender, and age or attempting to avoid stereotypes based on these characteristics. As a response to perceived discrimination, Black women shared concerns about appearing “angry,” “intimidating,” “attitudinal,” or “aggressive” in the workplace. Moreover, many mentioned avoiding appearing as an “angry Black woman,” specifically, and managing their behavior to avoid these stereotypes about Black women. Black feminist theorists and Black women studies scholars have written extensively about the roles of racist dominant stereotypes of Black women as mammies, matriarchs, welfare recipients, and sexually aggressive “Jezebels” as oppressive images Black women resist in society.^62–64^ Similarly, participants expressed the need to manage their behavior at work to avoid appearing too angry or emotional when dealing with perceived discrimination. Most participants declined to address experiences of discrimination at work due to fear of being stereotyped. Our findings are similar to studies of Black women experiencing work discrimination.^65–69^ Many of the participants described the need to appear present at work during pregnancy and postpartum to avoid stereotypes of laziness or slacking off work responsibilities. Additionally, many participants shared that they worked towards the end of pregnancy and returned promptly after their maternity leave.

Several studies have indicated that to negotiate the culture at work, Black Americans often must become bi-cultural, which means they must transition from their home/community culture to the White majority culture at work. Research by Walton^43^ with middle-class Black women found that members of this group have to negotiate Black culture and White culture constantly. Other scholars use the term “code-switching” or “shifting” to describe how Black Americans must navigate between different environments to succeed.^66,70^ Moreover, this requires Black women to modify their speech, hair, and dress at work.^27^ Often, these modifications are necessary to obtain employment, maintain employment, and command respect in the workplace. These modifications help Black Americans access a larger labor market but may cause harm to them by playing down their own racial and cultural identities at work. College education gives Black women wider access to a larger job market. The need to navigate work culture may be more salient for college-educated Black women in traditional “White collar” professional positions, especially those that are dominated by White men, such as corporate jobs. Annual 2023 reports by McKinsey & Company^71^ and Lean In^72^ indicated that Black women experience more microaggressions and less upward mobility, such as promotions in corporate jobs, than their White male counterparts. Furthermore, Black women in these occupations may experience more work-related stressors due to the pressures of maintaining these positions, especially as economic resources for their families.

Work-related stress emerged as a constant theme in Black women’s lives across the life course and did not change during pregnancy or postpartum. Furthermore, they reflected on mental and physical health responses to work-related stress. There is a dearth of research focusing on the impact of work-related stress and discrimination on Black maternal health (physical or mental). However, several studies have focused on the challenges Black women encounter in higher education as faculty and administrators.^73–75^ Similarly, several studies^25,44,65^ have examined how Black women navigate discrimination and cope in the workplace. These coping strategies include seeking support from co-workers or managers, family, faith and religious communities, identity shifting, avoidance, assimilation, and managing behavior.^27^ Moreover, Black women must cope with work-related discrimination throughout the life course, navigating the potential benefits and harms. Black women experience many psychological stressors over the life course, and the weathering hypothesis developed by public health scholar Arline Geronimus suggests that Black women’s health deteriorates as they age due to a lifetime of accumulated stress and over-coping related to this stress, which causes a childbearing disadvantage starting in young adulthood.^76,77^ Unequivocally, life course experiences coping with work-related stressors and discrimination have the potential to impact maternal health adversely. College-educated Black women have to address the stressors of navigating higher education that bring their own set of experiences of gender racism and stress. Moreover, these accumulated experiences need to be better understood and their impact on maternal health for this population of Black women.

BFT assumes that Black women experience and resist race, class, and gender oppression in the following spheres of their lives: on a personal level, on a group or community level, and on a systemic level.^20^ Furthermore, places of employment are institutions that are sites of domination and resistance for Black women.^20^ The context of the work environment and access to employment is shaped by interpersonal and structural oppression that Black women cope with and resist. Our findings align with the characteristics of the Superwoman Role found in Woods-Giscombe’s^40^ work on the SWS. These characteristics manifest strength, suppression of emotions, resisting vulnerability, and determination to succeed in the work environment. Our study participants also expressed similar perceived liabilities of the Superwoman Role, such as stress-related health behaviors (i.e. postponing healthcare) and the embodiment of stress.^38,41^ Participants expressed both mental and physical responses to work-related stress and discrimination that can lead to harmful health outcomes, especially adverse maternal health. Thus, in line with phenomenology’s emphasis on uncovering the essence of lived experiences, our study highlights how participants interpret their work-related stress and discrimination, not merely as external pressures but as deeply embodied experiences that shape their mental and physical health during and after pregnancy.

### Limitations

This study adds to the literature on psychological factors such as work-related stress and discrimination and how they influence experiences during pregnancy and postpartum for Black women. Limitations include the study participation being limited to Black women who had access to the internet and could complete the survey and in-person interview. Education is conflated with socioeconomic status; therefore, we do not know if Black women with higher education status but not middle income have similar workplace experiences or if there is a difference between women with only a bachelor’s degree versus higher graduate education. Additionally, potential bias may arise due to the reliance on self-reported data. Participants may face challenges in accurately recalling detailed information, especially when recalling situations over a long period.

## Conclusion

For college-educated Black women experiencing pregnancy and postpartum stages of pregnancy, it is crucial to understand the structural and social determinants of optimal occupational health. It is critical that workplaces enact occupational health equity to reduce gendered racism and ableist discrimination experienced by Black women during pregnancy and postpartum. Several studies have indicated disparities in access to paid leave for Black women. Therefore, paid leave equity could ease some of the stressors experienced by this group during postpartum. Policy interventions to address this social problem include taking advantage of Employee Assistance Programs and state-level policy changes to expand parental leave.

## Supplemental Material

sj-docx-1-whe-10.1177_17455057241304842 – Supplemental material for Work as a social determinant of maternal health: A qualitative exploration of college-educated Black women’s experiences at work during pregnancy and postpartum Supplemental material, sj-docx-1-whe-10.1177_17455057241304842 for Work as a social determinant of maternal health: A qualitative exploration of college-educated Black women’s experiences at work during pregnancy and postpartum by Serwaa S Omowale, Laurenia C Mangum, Andrea Joseph-McCatty, Cherell Cottrell-Daniels, Kaiya A Farris, Rashon King and Brittany C Slatton in Women’s Health

sj-docx-2-whe-10.1177_17455057241304842 – Supplemental material for Work as a social determinant of maternal health: A qualitative exploration of college-educated Black women’s experiences at work during pregnancy and postpartum Supplemental material, sj-docx-2-whe-10.1177_17455057241304842 for Work as a social determinant of maternal health: A qualitative exploration of college-educated Black women’s experiences at work during pregnancy and postpartum by Serwaa S Omowale, Laurenia C Mangum, Andrea Joseph-McCatty, Cherell Cottrell-Daniels, Kaiya A Farris, Rashon King and Brittany C Slatton in Women’s Health

## Declaration

**Ethics approval and consent to participate:** The University of Pittsburgh Research Office approved all study protocols and consent (IRB Number # STUDY1902029). All eligible participants provided verbal informed consent to participate in the Black Women’s Work and Maternal Health Study.

**Consent for publication:** The consent to participate includes consent to publish study findings. All data included in the manuscript is de-identified.

**Availability of data and materials:** Study data and materials are available upon request from corresponding author, SSO.
