# Supplementary material for: Work as a social determinant of maternal health: A qualitative exploration of college-educated Black women’s experiences at work during pregnancy and postpartum
Source: Womens Health (Lond). 2024 Dec 4;20:17455057241304842. doi: 10.1177/17455057241304842 (PMC11618909; doi:10.1177/17455057241304842)
Supplement: sj-docx-1-whe-10.1177_17455057241304842 – Supplemental material for Work as a social determinant of maternal health: A qualitative exploration of college-educated Black women’s experiences at work during pregnancy and postpartum [file sj-docx-1-whe-10.1177_17455057241304842.docx]

**Black Women’s Work and Maternal Health Research Study**

Interview Guide

1. What type of colleges or universities did you attend (e.g. PWI or HBCU)? Briefly, tell me

what your experience was like at that/those institution/s?

2. Have you experienced any incidents at work you believe are due to discrimination? (Do

you feel you experienced discrimination due to your race, gender or any other identity?)

How does this make you feel? How do you handle incidents like this at work?

3. What is the first thing you think about when you start a new job?

4. Can you describe how you present yourself to others at work? (Why is it important to

present yourself in this manner?)

5. How do you feel you are perceived by others at your workplace?

6. Can you tell me about a time you felt ill due to stress at work?

7. How do you usually cope or relax after a stressful day at work? (Can you provide some

specific examples)

8. Tell me about a time you did not seek medical attention due to work?

9. How would you describe your role in your family? How does this influence your work

experience?

10. Please describe your experience at work during the year prior to your last pregnancy?

How would you describe your experience at work during your most recently pregnancy?

11. Tell me how you feel you contributed to your health in a positive way during your most

recent pregnancy? How do you feel you contributed in a negative way?

12. How would you describe your health during your most recent pregnancy?

13. Please tell me about your experience during labor & delivery?

14. How would you describe your baby’s health at birth?

15. How would you describe your health immediately after giving birth (within the first 6

weeks)?

16. Please describe the support you received during your most recent pregnancy?

17. Did you take any time off related to your pregnancy? How long did you take off work?

18. Could you tell me about your experience returning to work after having your baby?

19. How would you describe the support you received when you returned to work. (Who

provided this support?)

20. What support did you need to transition back to work after your most recent pregnancy?
