# Supplementary material for: Work as a social determinant of maternal health: A qualitative exploration of college-educated Black women’s experiences at work during pregnancy and postpartum
Source: Womens Health (Lond). 2024 Dec 4;20:17455057241304842. doi: 10.1177/17455057241304842 (PMC11618909; doi:10.1177/17455057241304842)
Supplement: sj-docx-2-whe-10.1177_17455057241304842 – Supplemental material for Work as a social determinant of maternal health: A qualitative exploration of college-educated Black women’s experiences at work during pregnancy and postpartum [file sj-docx-2-whe-10.1177_17455057241304842.docx]

Table 1 Consolidated criteria for reporting qualitative studies (COREQ): 32-item checklist No Item Guide questions/description

**Domain 1:** Research team and reflexivity Personal Characteristics

1. Interviewer/facilitator Which author/s conducted the interview or focus group? Author, Serwaa S. Omowale conducted all interviews.

2. Credentials What were the researcher’s credentials? E.g. PhD, MD At the time of the interviews, Serwaa S. Omowale was a doctoral candidate at the University of Pittsburgh with an MSW.

3. Occupation What was their occupation at the time of the study? Serwaa S. Omowale was a doctoral candidate at the University of Pittsburgh.

4. Gender Was the researcher male or female? Female

5. Experience and training Relationship with participants. What experience or training did the researcher have? Serwaa S. Omowale was a clinically trained social worker with experience doing clinical and research interviews. Also, she had training in qualitative research at the master's and doctoral level from the University of Pittsburgh.

6. Relationship established Was a relationship established prior to study commencement? No

7. Participant knowledge of the interviewer What did the participants know about the researcher? e.g. personal goals, reasons for doing the research. The participants knew that Serwaa S. Omowale was a doctoral student at the University of Pittsburgh.

8. Interviewer characteristics What characteristics were reported about the interviewer/facilitator? e.g. Bias, assumptions, reasons and interests in the research topic. None

**Domain 2:** study design Theoretical framework

9. Methodological orientation and Theory What methodological orientation was stated to underpin the study? e.g. grounded theory, discourse analysis, ethnography, phenomenology, content analysis

Phenomenology was the methodological orientation.

Participant selection

10. Sampling How were participants selected? e.g. purposive, convenience, consecutive, snowball

Convenience snowball sampling

11. Method of approach How were participants approached? e.g. face-to-face, telephone, mail, email

Social media, email

12. Sample size How many participants were in the study?

17

13. Non-participation How many people refused to participate or dropped out? Reasons? Setting

None

14. Setting of data collection Where was the data collected? e.g. home, clinic, workplace

Workplaces, homes, public libraries (private rooms)

15. Presence of non-participants Was anyone else present besides the participants and researchers?

Some of the participants had their babies or toddlers with them during the interview.

16. Description of sample What are the important characteristics of the sample? e.g. demographic data, date

The study participants identified as Black women born in the United States.

Data collection

17. Interview guide Were questions, prompts, guides provided by the authors? Was it pilot tested?

Yes, the interview guide was provided to the authors. The questions were tested with other academics at the University of Pittsburgh.

18. Repeat interviews Were repeat interviews carried out? If yes, how many? No

19. Audio/visual recording Did the research use audio or visual recording to collect the data? Yes, a digital recorder.

20. Field notes Were field notes made during and/or after the interview or focus group? Yes, field notes were made.

21. Duration What was the duration of the interviews or focus group? The interviews last 20-60 minutes on average.

22. Data saturation Was data saturation discussed? Yes

23. Transcripts returned Were transcripts returned to participants for comment and/or correction? No

**Domain 3:** analysis and findings

Data analysis

24. Number of data coders How many data coders coded the data? 3

25. Description of the coding tree Did authors provide a description of the coding tree? No

26. Derivation of themes Were themes identified in advance or derived from the data? It was a deductive process based on theory.

27. Software What software, if applicable, was used to manage the data? DeDoose

28. Participant checking Did participants provide feedback on the findings? No

Reporting

29. Quotations presented Were participant quotations presented to illustrate the themes / findings? Yes

Was each quotation identified? e.g. participant number No

30. Data and findings consistent Was there consistency between the data presented and the findings? Yes

31. Clarity of major themes Were major themes clearly presented in the findings? Yes

32. Clarity of minor themes Is there a description of diverse cases or discussion of minor themes Yes
